# The Roles of microRNAs in Cancer Multidrug Resistance

**DOI:** 10.3390/cancers14041090

**Published:** 2022-02-21

**Authors:** Lucia Pavlíková, Mário Šereš, Albert Breier, Zdena Sulová

**Affiliations:** 1 Institute of Molecular Physiology and Genetics, Centre of Bioscience, Slovak Academy of Sciences, Dúbravská Cesta 9, 84005 Bratislava, Slovakia; lucia.pavlikova@savba.sk; 2 Institute of Biochemistry and Microbiology, Faculty of Chemical and Food Technology, Slovak University of Technology, Radlinského 9, 81237 Bratislava, Slovakia

**Keywords:** miRNA, multidrug resistance, ABC transporters, cell cycle, apoptosis

## Abstract

**Simple Summary:**

The resistance of neoplastic cells to multiple drugs is a serious problem in cancer chemotherapy. The molecular causes of multidrug resistance in cancer are largely known, but less is known about the mechanisms by which cells deliver phenotypic changes that resist the attack of anticancer drugs. The findings of RNA interference based on microRNAs represented a breakthrough in biology and pointed to the possibility of sensitive and targeted regulation of gene expression at the post-transcriptional level. Such regulation is also involved in the development of multidrug resistance in cancer. The aim of the current paper is to summarize the available knowledge on the role of microRNAs in resistance to multiple cancer drugs.

**Abstract:**

Cancer chemotherapy may induce a multidrug resistance (MDR) phenotype. The development of MDR is based on various molecular causes, of which the following are very common: induction of ABC transporter expression; induction/activation of drug-metabolizing enzymes; alteration of the expression/function of apoptosis-related proteins; changes in cell cycle checkpoints; elevated DNA repair mechanisms. Although these mechanisms of MDR are well described, information on their molecular interaction in overall multidrug resistance is still lacking. MicroRNA (miRNA) expression and subsequent RNA interference are candidates that could be important players in the interplay of MDR mechanisms. The regulation of post-transcriptional processes in the proteosynthetic pathway is considered to be a major function of miRNAs. Due to their complementarity, they are able to bind to target mRNAs, which prevents the mRNAs from interacting effectively with the ribosome, and subsequent degradation of the mRNAs can occur. The aim of this paper is to provide an overview of the possible role of miRNAs in the molecular mechanisms that lead to MDR. The possibility of considering miRNAs as either specific effectors or interesting targets for cancer therapy is also analyzed.

## 1. Introduction

In addition to the genotypic causes of cancer, phenotypic impulses also play an important role in their etiopathogenesis. Over the course of our lives, our physiology changes as we get older, and we are also exposed to many sometimes harmful environmental factors that can increase the incidence of cancer. A hectic modern lifestyle combined with an unbalanced diet with lower levels of protective and higher levels of harmful components, a polluted environment, stress exposure, low physical activity, the development of obesity, and many other factors that we encounter on a daily basis play a role in tumor induction and growth (reviewed in [1,2]). The incidence of cancer is constantly growing and growing faster than the human population [3]. The incidence of new cancer cases was projected to increase from 12.7 million per year in 2008 to 22.2 million per year in 2030 [4], but the population is growing more slowly [5].

The treatment of cancer depends on the type, location, degree of malignancy of the tumor, and the condition and age of the patient. Currently, the most common treatments are surgical removal of tumor tissue, radiotherapy, and chemotherapy combined with immunotherapy [6]. Chemotherapy is used as an adjunctive treatment for solid tumors and can be applied before and/or after surgery. In hematooncological diseases, in addition to allogeneic [7,8] or autologous [9] bone marrow cell transplantation, intensive chemotherapy is often the only possible treatment. However, a portion of patients (non-responders) do not respond to chemotherapy from the beginning [10]. Other patients who initially respond to chemotherapy in remission may lead to treatment resistance and relapse upon repeated administration of cytostatics [11]. Researchers often encounter resistance not only to the substance being administered, but also to other substances with different structures and mechanisms of action, which is known as multidrug resistance (MDR, reviewed in [12]). Although considerable progress has been made in the development of chemotherapeutics in recent decades, MDR remains a major obstacle to successful treatment [13].

Biological processes in the cell are controlled by the precise multilevel regulation of gene expression. Regulatory checkpoints for gene expression include regulation of transcription; splicing of precursor pre-mRNA into coding mRNA; transport of mRNA across the nuclear envelope; RNA-mediated post-transcriptional silencing; regulation of translation; post-translational modifications of proteins and protein folding [14]. Post-transcriptional processes leading to translation are significantly attenuated by small forms of RNA, including miRNAs. MiRNAs are endogenous noncoding 17–24-base oligoribonucleotides that result from the cleavage of double-stranded (ds) precursor microRNAs (pre-miRNAs) and are partially complementary to target mRNAs [15]. They can attack mRNA molecules based on their complementarity and prevent them from binding to the ribosome. In addition, miRNAs form complexes with partner proteins (to form the RNA interference silencing complex (RISC)), some of which have endonuclease activity [16]. Such a complex bind to mRNA destabilizes it and induces its degradation [17]. Thus, the miRNA suppresses the level of translation from the target mRNA. In this way, miRNAs affect the overall cellular machinery, including cell development, cell division, cell cycle and growth, cell differentiation, cellular responses to stress, whole cellular metabolism, cellular responses to immune signals, and the cellular ability to initiate apoptosis [18,19]. In humans, more than 1000 miRNAs are thought to regulate the expression at the post-transcriptional level of at least 60% of genes throughout the genome [20]. Genes for miRNAs represent approximately 3–4% of all human genes, making them one of the largest classes of gene regulators [21]. Functional miRNAs bind to RNA-induced silencing complex (RISC) forms with partner proteins, of which proteins from the argonaut family (AGO) play a central role [22]. Although members of the argonaut family (AGO1, AGO2, AGO3, and AGO4 in humans) are able to load miRNA, endonuclease activity and thus mRNA cleavage belong exclusively to AGO2 [23,24]. Usually, the binding of miRNAs to mRNAs does not affect protein production more than twofold [25]. In the case of mRNA molecules that have multiple miRNA binding sites, the binding effects of the individual miRNAs could be amplified with each other and could produce up to a 10-fold suppressive effect. The amount of suppression or induction that miRNA induces also depends on the level of its expression. It could also influence a group of genes acting in the miRNA pathway [26]. Much more information on miRNA biology has been included in the review article by Bartel [27], and therefore we do not consider it necessary to address it further. 

**Figure 1:**
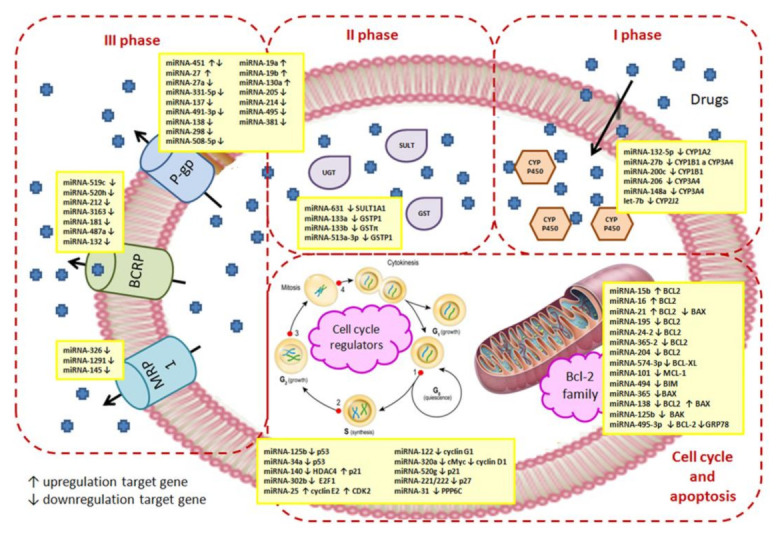
MiRNAs involved in the regulation of MDR mechanisms. The natural effect of miRNAs is to suppress translation from the respective mRNA, which is either blocked or subject to degradation. However, upregulation of proteins may also occur, e.g., proto-oncogenes, if the miRNA is targeted to tumor suppressor genes. In MDR, miRNAs target phases I, II, and III of detoxification and are also active in systems regulating the onset and progression of apoptosis or cell cycling.

Different lines of evidence suggest that there exists a multilevel interplay between the regulatory effects of miRNAs and the development of MDR [28,29,30]. In the current paper, we have summarized the information on the various relationships between the mechanisms leading to MDR and the expression/function of some miRNAs. Many mechanisms of MDR are currently well known [11,12], of which we will address the elevated expression of either ABC transporters or drug-metabolizing enzymes, impaired programmed cell death, particularly apoptosis, and the changes in cell cycle checkpoints. A summary of miRNA effects is given in Figure 1 and is also documented in Table S1 in the supplementary data for better orientation.

## 2. Cell Resistance to Multiple Drugs

In the course of evolution, living organisms have developed multiple mechanisms of response to harmful chemicals present in the environment [31]. Activation of the cell’s defense pathways subsequently leads to an increase in the resistance of cells to various chemicals [32], often with fundamentally different structures and mechanisms of action, which is called multidrug resistance [11]. The development of drug resistance, when present in neoplastically transformed human cells, is a real obstacle to anticancer treatment [12]. Processes leading to multidrug resistance may involve several mechanisms, including the following [33]: (i) changes in the content and function of pharmacological drug targets; (ii) increased drug efflux through the overexpression of plasma membrane transporters; (iii) modification of drugs by metabolizing enzymes; (iv) acceleration of cellular repair mechanisms, including DNA repair; (v) changes in the regulation of cell death programs; and (vi) changes in proliferation regulation and the cell cycle. Drug modification and removal of parent drugs or their metabolites from neoplastic cells play an important role in cellular protection against chemical stress [34]. Xenobiotic detoxification involves a step-by-step mechanism (detoxification phases I–III [35]). Transforming enzymes of the first phase alters compounds by oxidation, reduction, or hydrolysis to make them more soluble in urine and readily excretable. These reactions are mediated by versatile cytochrome P450s (CYP) enzymes and more selective flavin-containing monooxygenases and monoamine oxidases [36]. CYPs are heme-containing proteins and are members of one of the largest protein families (consisting of 57 genes in humans) [37]. Either parent compounds or CYPs-altered compounds may be further subjected to conjugating enzymes in the process of glucuronidation, acetylation, sulfation, glutathione conjugation, acetylation, aminoacyl conjugation, or methylation. These processes adjust toxic compounds as substrates for transporters (predominantly from the ABCC subfamily) that ensure their transport out of the cell [35,38]. 

## 3. The Role of microRNAs in the Development of Neoplastic Cell Resistance to Chemotherapy

It is generally accepted that in addition to changes in the genome, the regulation of gene expression is disrupted in cancer cells, leading to changes in the transcriptome and proteome profiles [39,40]. In addition to changes in the transcription of gene encoding proteins, there are also changes in transcription from genes providing noncoding RNAs, including miRNAs. If a change in the expression of gene encoding proteins makes it possible to deduce from the knowledge of their function and position in metabolic and/or regulatory pathways what change in cellular homeostasis occurs, the situation is more complicated by changes in miRNA expression. As mentioned above, individual miRNAs can elicit responses in regulating the expression of multiple genes [41], which are often involved in various metabolic and regulatory functions of cells. In several cases, this makes it impossible to easily predict what changes in the expression levels of individuals miRNAs cause in particular cells. In addition, miRNAs regulate various genes that affect the response of cells to chemotherapeutics [42]. It should be appreciated that the regulation of miRNAs may be specific for the tissue/cell and condition in which they are expressed, as well as the drug used for chemotherapy. Thus, the same miRNA could induce different responses in different tissues/cells in general and, of course, in regulating the response of cells to anticancer drugs. For this reason, we may sometimes encounter illusive controversy in studies describing the effects of miRNAs on the sensitivity of cancer cells to drugs.

According to their relation to cancer progression, miRNAs are divided into oncogenic and tumor suppressors [43,44]. Oncogenic miRNAs promote and are overexpressed in tumors and, in particular, cause suppression of tumor suppressor gene expression (Supplementary files, Figure S1). In contrast, tumor suppressor miRNAs have reduced expression in tumors, resulting in the suppression of their ability to inhibit tumor growth by eliminating the expression of oncogenes and genes regulating cell differentiation and the onset/progression of apoptosis [45].

The miRNA-17-92 cluster, which is located in the MIR17HG intron on the q arm of chromosome 13 at position 31.3, provides approximately 0.8 kb of pri-miRNA-17-92 [46]. This miRNA is further processed into seven different mature miRNAs: miRNA-17-5p, miRNA-17-3p, miRNA-18a, miRNA-19a, miRNA-19b, miRNA-20a, and miRNA-92a. These miRNAs are important negative regulators of the expression of tumor suppressor genes such as PTEN (phosphatase and tensin-like protein), TGFBR2 (transforming growth factor beta receptor 2), SMAD4 (mothers against decapentaplegic homolog 4), and p21 (cyclin-dependent kinase 2 inhibitor). The antiapoptotic effect of miRNAs derived from the miRNA-17-92 cluster accompanied by the promotion of cell proliferation and growth was also confirmed [47], and the mediating effect on tumor angiogenesis was stimulated by the activation of oncogenic c-Myc (a protooncogenic transcription factor) [48]. In addition, these miRNAs control cellular chemoresistance and increase tumor growth in mantle cell lymphoma through PI3K/AKT acceleration [49]. The oncogenic family of miRNAs also includes miRNA-155, which is associated with increased expression of oncogenic MYC in B-cell transformation [50]. This miRNA is a key regulator of glucose metabolism by controlling the PIK3R1-PDK/AKT-FOXO3a-cMYC axis [51].

Genome-wide screening of genes that are miRNA-21 targets has shown that miRNA-21 overproduction deregulates the expression of genes whose products are active either at cell cycle checkpoints or in the DNA damage response [52]. Both of these functions could have an impact on the acceleration of cell growth mediated by RAS [53]. Furthermore, miRNA-21 reduces apoptosis, thereby also potentiating tumor cell growth [54]. Oncogenic miRNA-21 negatively regulates PTEN and activates phosphoinositide-3 kinase (PI3K)/AKT [55]. MiRNA-21 promotes chemoresistance in many types of tumors. Different levels of miRNA-21 expression in the MCF-7 cell line affect its sensitivity to doxorubicin [56]. Upregulation of miRNA-21 is accompanied by downregulation of PTEN. However, PTEN, as a tumor suppressor, reduces the resistance of cells to doxorubicin. Thus, overexpression of miRNA-21 increases doxorubicin resistance by reducing the PTEN content of these breast cancer cells [56]. This miRNA also increases doxorubicin resistance in bladder cancer-derived T24 cells [57]. In these cells, miRNA-21 induced increased proliferation and resistance to doxorubicin accompanied by upregulation of BCL-2, which inhibited entry into doxorubicin-induced apoptosis. Upregulation of BCL-2 induced by miRNA-21 was suppressed by the PI3K inhibitor LY294002. However, a direct relationship between inhibition of PI3K and suppression of miRNA-21-induced doxorubicin resistance may not exist because this inhibitor antagonizes the expression/activity of the P-glycoprotein (the most commonly occurring drug plasma membrane transporter, known as ABCB1 or P-gp, see Section 4.3.1) [58], of which doxorubicin is a known substrate. One of the most effective drugs for the treatment of leukemia is daunorubicin (DNR) [59]. In the DNR-resistant cell line K562/DNR, the expression of miRNA-21 was increased compared to that in the sensitive parental line K562. Stable transfection of miRNA-21 in K562 cells led to the development of resistance to DNR, while suppression of miRNA-21 in K562/DNR cells led to increased cytotoxicity of DNR.

In colorectal carcinoma cells, miRNA-21 inhibits 5-FU (fluorouracil)-induced cell arrest in the G2/M phase of the cell cycle. In vivo experiments have shown that overexpression of miRNA-21 reduces the therapeutic effect of 5-FU and induces resistance to it in mice [58,60]. In contrast to miRNA-21, miRNA-137 reduces the resistance of MCF-7 cells to doxorubicin by upregulating Y-box-binding protein-1 (YB-1) and subsequently upregulating P-gp [61]. In contrast, transfection of P-gp-positive leukemia cells with miRNA-27a mimic led to a decrease in cellular P-gp content [62]. Similarly, transfection of bladder cancer cells with miRNA-27a mimic leads to the downregulation of P-gp, which appears to be related to the FZD7 (frizzled class receptor 7)/β-catenin pathway [63]. However, an increase in P-gp expression was achieved after transfection of human A2780 ovarian cells with miRNA-27a mimic [64]. This points to the possibility that miRNA-27a may have opposite effects in different tumor cells. In addition, this miRNA modulates cisplatin resistance in bladder cancer by targeting the cystine/glutamate exchanger SLC7A11 [65].

Elevated levels of other miRNAs have also been observed in chemoresistant breast cancer cells, e.g., miRNA-203, miRNA-125b, miRNA-34a, and miRNA-663. Their inhibition may partially restore the sensitivity of cancer cells to drugs, e.g., tamoxifen, docetaxel, 5-FU, and cisPt [66,67,68,69]. All of the above findings suggest that miRNAs represent a potential target in the treatment of chemoresistant tumors and that their modulation in combination with chemotherapeutics has the potential for use in anticancer therapy.

Sorafenib is a multityrosine kinase inhibitor [70] that can directly or indirectly inhibit tumor growth. Its primary target is receptor tyrosine kinases, which play crucial roles in cell growth, metabolism, differentiation, and motility [71]. Sorafenib is used to treat otherwise incurable neoplastic diseases, such as advanced renal cell carcinoma, especially in patients who have failed or are considered unsuitable for previous interferon-alpha or interleukin-2 therapy [72]. However, resistance to sorafenib may occur. Mao et al. [73] reported that the IC_50_ value for sorafenib is significantly higher for hepatocellular carcinoma (HCC) cells infected with hepatitis B virus than for uninfected cells. The concomitant phenomenon of this infection in HCC cells was the downregulation of miRNA-139b. Restoration of miRNA-139b expression after transfection with the miRNA-193b mimic increased the sensitivity of HCC cells to sorafenib.

The most common mechanism of drug resistance is considered to be the elimination of drugs from the intracellular space mediated by membrane transporters, especially from the ABC family [12,74,75]. In addition to the above miRNA-137 and miRNA-27a, miRNA-223 is also targeted to P-gp [76]. The gene for another multidrug resistance protein 1 transporter (MRP1, ABCC1 transporter) contains a potential miRNA binding site for miRNA-133a and miRNA-326 on the 3′-untranslated region [77]. A significant decrease in the transcription level of MRP1 was observed after transfection of HepG2 hepatocellular carcinoma cells with miRNA-133a and miRNA-326 mimic and was also accompanied by a decrease in MRP1 immunoreactivity on western blots. Cisplatin is often used in the first regimen to treat several types of cancer originating from the ovaries, the lungs, the head and neck of the uterus, the testicles, or the bladder [78]. It is effective in the treatment of germ cell tumors, sarcomas, carcinomas, lymphomas, and others. A wide range of miRNAs, including let-7c [79], miRNA-31 [80], miRNA-138 [81], miRNA-182 [82], miRNA-205 [83], miRNA-224 [84], miRNA-106a [85], miRNA-15b [86], miRNA-27a [87], miRNA-513a-3p [88], miRNA-34a [89], and miRNA-92b [90], regulate the tolerance of non-small cell lung cancer to cisplatin.

ABCC2 is a membrane transporter that increases the outflow of drugs and reduces their intracellular concentration. Overexpression of ABCC2 can induce tumor cell resistance to a number of drugs, including cisplatin. BCL-XL is a member of the BCL-2-family of antiapoptotic proteins that help inhibit chemotherapeutic-induced apoptosis. Let-7c is able to simultaneously target ABCC2 and BCL-XL and reduce their expression, thereby increasing the sensitivity of A549 cells to cisplatin [79]. In addition, miRNA-31 [81] and miRNA106a [85] inhibit the translation of the ABCB9 and ABCA1 transporters, respectively. Both miRNAs reduce the sensitivity of non-small cell lung cancer cells to cisplatin.

Oncogenic miRNA-302s (miRNA-302a-3p, miRNA-302b-3p, and miRNA-302c-3p) are upregulated in NT2-D1 and 833 K tumor germ cell lines, and treatment with cisplatin suppresses this upregulation [91]. These miRNAs appear to control the cellular contents of SPRY4-mitogen-activated protein kinase (MAPK) signaling pathway inhibitors, which regulate the MAPK/ERK and PI3K/Akt signaling pathways in these cells.

Oncogenic miRNA-21 was found to be upregulated when the A2780CP20 variant of the cisplatin-resistant ovarian carcinoma cell line was compared to the cisplatin-sensitive A2780 counterpart [92]. The presence of c-Jun attached to the miRNA-21 region of the promoter was demonstrated by a chromatin immunoprecipitation assay.

Huang et al. [66] identified miRNA-650 expression as an independent prognostic factor for predicting the survival of patients with lung adenocarcinoma (LAD) and found that miRNA-650 levels in LAD tissue correlated with patients’ response to docetaxel. However, in vitro experiments showed that silencing miRNA-650 reversed the resistance of cell lines derived from the original LAD by selection for docetaxel. Upregulation of miRNA-650 reduced the sensitivity of sensitive LAD cells to docetaxel, both in vitro and in vivo [66]. MiRNA-10b appears to mediate resistance to 5-FU by downregulating the proapoptotic protein BIM in colorectal cancer cells [93]. Increased expression of miRNA-20a through downregulation with BNIP2 (BCL2/adenovirus E1B 19 kDa protein-interacting protein 2) is associated with colorectal cancer cell resistance to fluorouracil, oxaliplatin, and teniposide (Chai et al. [94]). Deletion of miRNA-20a reduced their resistance to these chemotherapeutics, while the overexpression of miRNA-20a in cells increased their chemoresistance.

Boyerinas et al. [95] found that members of the let-7 family target IMP-1 (insulin-like growth factor mRNA binding protein 1), which prevents translation of mRNA for the P-gp transporter, thereby increasing the sensitivity of cells to its substrates, including Taxol. Insertion of let-7g into DOX-RES cells expressing both IMP-1 and P-gp resulted in decreased expression levels of both proteins. The cells were thus more sensitive to taxol and vinblastine without affecting their sensitivity to carboplatin, which is not a substrate of P-gp.

## 4. Effects of miRNAs on Cell Detoxification Pathways

**Figure 2:**
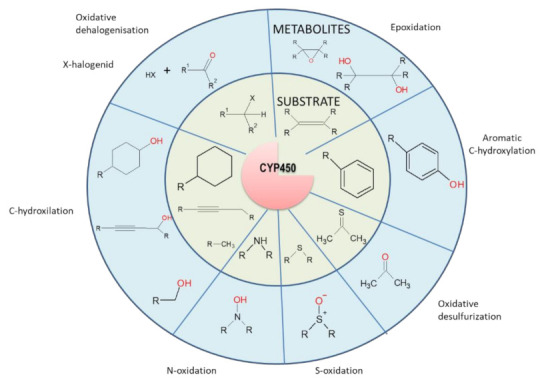
Examples of cytochrome P450-catalyzed reactions. More details were described in Rydberg et al. [103].

**Figure 3:**
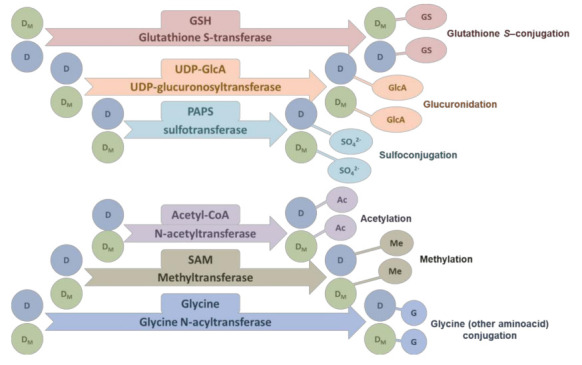
Typical reaction catalyzed by conjugation enzymes. More details were described in Jančová et al. [125]. Abbreviations: GSH–reduced glutathione, UDP-GlcA–uridine diphosphate glucuronic acid, PAPS–3′-phosphoadenosine-5′-phosphosulfate, SAM–S-adenosyl-l-methionine.

Systems that provide drug metabolism and elimination in cellular detoxification protection can be activated in tumor cells and play an important role in the development of cell resistance to various drugs [96]. The metabolism of drugs of a lipophilic nature consists of three phases: phase 1–oxidation, realized mainly by cytochrome p450; phase 2–conjugation, realized by conjugation of enzymes such as glutathione S-transferases, UDP-glucuronyl transferases and others; phase 3–transport, realized by membrane transporters [97] (Figure 1). In the first phase, the polarity of the lipophilic substance is increased by introducing hydrophilic groups (most often -OH) into the molecule (Figure 2). In the second phase, the drug is conjugated to endogenous intermediates (glucuronic acid, glutathione, amino acids such as glycine, or inorganic anions such as sulfate; Figure 3). The conjugation products formed in this way are more easily secreted because the added anionic moiety directs them to bind and transport by membrane anion transporters (Figure 1). In the third phase, substances or their metabolites are secreted from the cell by transporters predominantly from the ABC family [98]. MiRNAs, due to their ability to regulate a wide range of genes, including almost all mechanisms of MDR (Figure 1), could be biomarkers for the reliable prediction of cell responses to chemotherapy, and/or a potential target for overcoming MDR.

### 4.1. The First Phase of Drug Metabolism–Oxidation

Key enzymes in the first phase of drug metabolism belong to the family of cytochrome P450 (CYP) enzymes, which catalyze oxidoreductive reactions of substances (either endogenous intermediates and end products or xenochemicals, including drugs; Figure 2) [99]. These cytochromes are heme-containing oxidoreductases (Fe chelated in a porphyrin backbone) that utilize the oxidation stages (2+/3+) of central Fe for electron acceptance/transfer, and as electron-donating cofactors, they use nicotinamide adenine dinucleotide (phosphate) [100]. In humans, the predominant amount of CYPs is attached to the membrane of the endoplasmic reticulum due to the transmembrane peptide anchor in its structure [101]. These enzymes are able to metabolize substrates coming from the cytosol as well as from the membrane environment, as their active sites are supplied by a complex network of access channels. The following members of four subfamilies perform a predominant number of reactions: 1A, 2C, 2D, and 3A. Moreover, in humans, 13% of common chemicals and 27% of drugs are metabolized by one member, CYP3A4 [102].

In the case of toxic stress, CYP expression in cells is often induced by xenosensing receptors: pregnane X receptor (PXR), constitutive androstane receptor (CAR), and peroxisome proliferator-activated receptor α (PPARα), which bind to DNA as heterodimers with retinoid X nuclear receptor; or the aryl hydrocarbon receptor (AHR), which dimerizes with hypoxia inducible factor β (HIFβ) [104]. Many exogenous substances are ligands for these receptors, including environmental pollutants and drugs.

However, CYP expression can be attenuated in cells by multiple miRNAs, as shown in Figure 1. Many CYP members, including three CYP1, seven CYP2, and CYP3A4, are directly targeted by multiple miRNAs, with experimental in vitro confirmation in more than 30 studies (as of August 2018) [105].

CYP-mediated drug metabolism can lead to either suppression or activation of their biological effects and even to the emergence of a new effect due to the ability of the modified drug to hit another biological target. Thus, CYP metabolism may suppress, potentiate, or alter the effects of the administered drug.

The following are some examples of the effects of miRNAs on the expression of members of the CYP family.

CYP1A2 is one of the most important enzymes involved in drug metabolism; its expression in the liver reaches 13–15% of all CYPs [106], and it is metabolized by approximately 9% of currently prescribed drugs [107]. Several polymorphisms of the CYP1A2 gene are associated with an increase in its drug-metabolizing activity and a worsening of the prognosis of, for example, lung cancer [108,109]. Its expression is effectively reduced by miRNA-132-5p CYP1A2 in hepatocytes, and this reduction is alleviated by lansoprazole-enhanced hepatotoxicity induced by flutamide [110]. Suppression of the presence of CYP1A2 decreases drug metabolism and consequently reduces lansoprazole/flutamide drug–drug interactions.

CYP1B1 metabolizes some pro-carcinogens, such as polycyclic aromatic hydrocarbons or 17-β-estradiol [111,112]. CYP1B1 is post-transcriptionally downregulated by miRNA-27b. Decreased miRNA-27b expression and consequently increased CYP1B1 expression may be one of the causes of tumor cell resistance to docetaxel [113,114]. Mu et al. have shown [115] that overproduction of miRNA-27b can increase the sensitivity of cancer cells to a broad spectrum of cytostatics by activating p53-dependent apoptosis and reducing CYP1B1-mediated detoxification. MiRNA-200c is involved in the regulation of CYP1B1 in renal cancer cells and docetaxel resistance [116]. Using several renal cancer cell lines, miRNA-200c has been shown to directly target CYP1B1, which is overexpressed upon downregulation of this miRNA.

Mao et al. [117] detected reduced levels of miRNA-187-5p in non-small cell lung cancer. The direct target appears to be CYPB1. The expression level of miRNA-187-5p reciprocally correlates with a good disease prognosis defined by the TNM stage and predicts the extent of postoperative survival. Overexpression of miRNA-187-5p correlates with tumor growth and metastasis.

Transfection of HepaRG cells with miRNA-130b mimic reduced overall CYP activity by 30% [118]. Furthermore, decreases in the expression levels of CAR, farnesoid X receptors and CYPs (members 1A1, 1A2, 2A6, 2C8, 2C9, and 2C19) as well as glutathione S transferase α2 (GSTA2) were found. However, the authors did not explicitly emphasize the link between their results and drug resistance.

CYP3A4 is a predominant CYP (20–30% in the liver) and metabolizes approximately 30–37% of commonly prescribed drugs [102,107]. For this reason, much attention is given to its study. MiRNA-27b levels may appear to affect CYP3A4 levels/activity in vivo via blockade of translation rather than affecting mRNA levels [119]. Overexpression of human miRNA-27b or mouse micro-RNA298 (mmu-miRNA298) in LS-180 and PANC1 cell lines reduced CYP3A4 expression by more than 30% and at the same time reduced the sensitivity of cells to cyclophosphamide [120]. Liu et al. [121] showed that miRNA-27b may also affect the metabolism of atorvastatin (a blood cholesterol-lowering medicine) in the liver. Nevertheless, statins are not commonly accepted as anticancer drugs, and Jones et al. [122] have shown that statins affect proliferation and metastasis in ovarian cancer. Atorvastatin inhibited cancer cell proliferation (in a dose-dependent manner), and its activity was associated with the induction of apoptosis, autophagy, cell stress, and cell cycle arrest via the AKT/mTOR and MAPK pathways. Moreover, atorvastatin also altered cell adhesion and invasiveness and reduced the expression of VEGF and MMP9, which are important for epithelial–mesenchymal transition and angiogenesis [122]. Overexpression of miRNA-148a reduces PXR protein levels, thereby attenuating CYP3A4 mRNA induction [123]. MiRNA-206, which suppresses CYP3A4 expression and activity by directly binding to the 3′-UTR of CYP3A4 mRNA, may also affect CYP3A4 metabolism and regulation [121].

Downregulation of CYP contents by miRNAs could exert direct effects on the characteristics of cancer tissue. The following example documented this possibility. Detection of the level of transcription with the luciferase reporter plasmid containing the 3′-UTR of CYP2J2 cloned downstream of the luciferase reporter gene transfected together with let-7b showed a reduction in expression [124]. Consistently lower CYP2J2 protein levels determined by western blotting were found after application of let-7b to HeLa, Tca-8113, SKMES-1, and MDA-MB-435 cell lines. Simultaneously, a reduction in cell proliferation and promotion of cell apoptosis were observed under CYP2J2 suppression [124].

### 4.2. The Second Phase of Drug Metabolism–Conjugation

The second-phase enzymes bind various side chains to the parent substances or substances modified during the first phase of drug metabolism. There are several transferases active in drug modification, the most important of which catalyze the reactions summarized in Figure 3 [125].

While conjugation with glutathione, glucuronidation, and sulfoconjugation increase the hydrophilic properties of the resulting conjugate, methylation and acetylation tend to improve the hydrophobic properties. A special case is conjugation with amino acids, where the contribution of the amino acid to the hydrophobic/hydrophilic properties is conditioned by the nature of the amino acid residue. It is generally believed that the collective activity of the enzymes of the first and second phases of detoxification results in an increase in the hydrophilicity of the metabolite, leading to increased urinary and fecal excretion [126]. Therefore, in the following, we will focus on conjugation reactions leading to the enhancement of the hydrophilic properties of conjugates.

These include sulfotransferase (products of *SULT* genes), glutathione transferase (products of *GST* genes), and UDP-glucuronosyltransferase (products of *UGT* genes). There is extensive knowledge about the modulation of enzymes of these three families with miRNAs (reviewed in [127,128]). Below are some examples of such regulation.

The SULT family of genes encodes sulfotransferases that catalyze the transfer of a sulfonyl group from PAPS (3′-phosphoadenosine 5’-phosphosulfate, see Figure 3) to the nucleophilic groups of various xenobiotics and endogenous compounds, thereby increasing their solubility and excretion [129].

The variability of SULT1A1 activity influences the efficacy of some drugs, and consequently induces drug resistance [130]. In vitro assays for promoter activity using luciferase as a reporter confirmed that the direct target of miRNA-NA-631 is SULT1A1 in a genotype-dependent manner [131]. The authors transfected the ZR75-1, MCF7, and MCF10A cell lines with the miRNA-631 inhibitor. The miRNA-631 inhibitor increased both the mRNA and protein products of the SULT1A1 gene in MCF7 and MCF10A cells but not in ZR75-1 cells [131]. Differences in SULT1A1 expression in regulation with miRNA-631 depended on the presence of SNP variant alleles in the 3′-UTR that are not present in ZR75-1. 

GSTP1 belongs to a family of glutathione transferases that catalyze the conjugation of electrophiles with glutathione (Figure 3), including platinum drugs such as cisplatin and carboplatin [132]. GSTP1 is involved in stress responses, cell signaling, and apoptosis, and its deletion selectively affects cisplatin and carboplatin chemosensitivity, cell invasiveness, and the ability to migrate [133]. Increased GSTP1 expression was observed in L1210 variants of cells expressing the P-gp transporter [134], which are less sensitive to cisplatin than the parental cells [135], although cisplatin is not a substrate of the P-gp transporter. GSTP1 overexpression has been observed in many types of cancer. Moriya et al. [136] showed that decreased miRNA-133a expression leads to an increase in GSTP1 and contributes to drug resistance. Overexpression of miRNA-133a suppressed GSTP1 expression at both the mRNA and protein levels in various cell lines was observed. Expression of miRNA-133a inhibited the proliferation, invasion, and migration of cancer cells, suggesting that it may function as a tumor suppressor. MiRNA-513a-3p may negatively regulate GSTP1 expression. Expression of this miRNA increased the sensitivity of CisPt-resistant A549 cells to cisplatin [88].

The UGT1A subfamily is involved in the metabolism of more than 50% of drugs, including irinotecan, raltegravir, and mycophenolic acid, which are used to treat cancer, HIV, and organ transplant rejection. The regulation of UGT1A involves miRNA-21-3p, miRNA-141-3p, and miRNA-200a-3p [137]. However, the authors did not address the issue of drug resistance. The fact that these miRNAs regulate several enzymes of the UGT family makes them interesting possible targets in supportive therapy [138].

The UGT2B subfamily is responsible for the modification of some endobiotics (bile acids and steroid hormones) and xenobiotics (cytostatics) [139]. Dluzen et al. [140] showed that miRNA-216b-5p can affect the efficacy of epirubicin treatment of liver cancer by targeting UGT2B4 and UGT2B10. In addition, UGT2B4 may also be regulated by miRNA135a [141], although the authors have not shown an association with drug resistance in cancer cells.

### 4.3. The Third Phase of Drug Metabolism-Transporters

Cellular efflux of drugs and their metabolites produced by first- and second-phase detoxification enzymes is one of the most studied mechanisms of multidrug resistance in cancer cells. There are several families of membrane transporters that, when overexpressed, can provide neoplastic cells with protection against the devastating effects of chemotherapeutics [142]. Members of the ABC transporter family (according to the presence of a conserved ATP-binding cassette [143]) represent the largest and most researched family of membrane transporters, which are jointly responsible for the development of MDR. MDR may be associated with overexpression of one or more ABC transporters [144]. The human genome includes 49 genes that contain a typical ABC motif in structure [145]. These genes, with a few exceptions, encode ATP-driven membrane pumps and play an irreplaceable role in the membrane transport of substances [145]. In particular, the following three proteins represent the generally accepted molecular causes of MDR development (reviewed in [12,74,96,143]): (i) permeability glycoprotein (P-glycoprotein), which is the first member of the B subfamily of ABC genes (also known as ABCB1, MDR1, and P-gp); (ii) breast cancer resistance protein, which is a second member of the G subfamily of ABC genes (also known as ABCG2, mitoxantrone transporter, and BCRP); (iii) multidrug resistance-associated protein 1, which is the first member of the C subfamily of ABC genes (also known as ABCC1 and MRP1). In an effort to avoid confusion, these proteins are referred to as P-gp, MRP1, and BCRP throughout the paper.

#### 4.3.1. Permeability Glycoprotein P-gp/ABCB1/MDR1

P-glycoprotein was the first ABC transporter to be discovered as a unique entity in MDR neoplastic cells and is responsible for altered permeability to some drugs [146,147]. P-gp consists of two halves on a single polypeptide chain, each of which contains one ATP-binding site with ABC consensus and one transmembrane domain consisting of six α-helical spans (reviewed in [12]). P-gp is currently the most frequently detected member of the ABC transporter family in MDR cancer tissue. This protein confers cellular resistance to a broad but well-defined group of drugs (P-gp substrates), including taxanes (paclitaxel and docetaxel) [148], epipodophyllotoxin derivatives (etoposide and teniposide) [149], anthracyclines (doxycyclines) [149,150], antibiotics (actinomycin D) [149,151], vinca alkaloids (vinblastine and vinorelbine) [152], and tyrosine kinase inhibitors (imatinib and dasatinib) [153]. Several lines of evidence suggest that P-gp may also be involved in the development of secondary resistance to P-gp nonrelated substances and that this resistance is independent of its P-gp drug-transport activity (reviewed in [12]). Therefore, we also observed less pronounced resistance to cisPt [135], tunicamycin [154], thapsigargin [155], and even lectin concanavalin A [156,157] in P-gp-expressing cells. In addition to the examples of the effects of miRNAs on P-gp regulation described in Section 3, others are provided here. MiRNA-19a/b are overexpressed in gastric cancer cell lines with the MDR phenotype, and these miRNAs increase P-gp expression by targeting PTEN (as Akt phosphorylation inhibitor) [158]. MiRNA-451, miRNA-27a, and miRNA-130a are active in human cancer cells, where they upregulate P-gp [64,159].

The regulation of P-gp expression described in further studies points to significant variability in the results and the fact that the response to miRNAs may vary depending on the nature of the cells and their current condition. Imatinib mesylate (IM) resistance induced by previous treatment of patients with chronic myeloid leukemia was accompanied by decreased miRNA-214 content and increased P-gp expression [160]. Increased miRNA-214 levels restored IM sensitivity in resistant cells accompanied by altered P-gp expression. [160]. The same effect in suppressing P-gp expression was observed when miRNA-205 was transfected into docetaxel-resistant prostate cancer cells, where its level is naturally suppressed [161]. In colorectal cancer, a decrease in miRNA-26b expression in 5-FU-resistant cells was observed, and the authors found that its promoter was hypermethylated and that its higher expression was observed only in susceptible cells [162]. Kovalchuk et al. [163] found increased expression of P-gp, antiapoptotic protein BCL6 (B-cell CLL/lymphoma 6), and NOTCH1 (human single-pass transmembrane receptor with functions in cell differentiation) in doxorubicin-resistant MCF7/DOX cells compared to parental MCF7 cells. In the resistant cell line, the expression level of miRNA-451 was below the limit of detection. The authors confirmed the negative regulatory effect of miRNA-451 on P-gp expression using 3′-UTR gene assays [163]. In addition, transfection of MCF7/DOX miRNA-451 cells restored cell sensitivity to doxorubicin. A similar phenomenon has been observed in leukemic cells and hepatocellular carcinoma cell lines [63,164], suggesting that cell line type and environment may affect the role of miRNA. MiRNA-331-5p and miRNA-27a reciprocally correlate in the K562 cell line with the expression of P-gp, which confers resistance to doxorubicin [164]. Transfection with miRNA-331-5p or miRNA-27a (alone or in combination) in doxorubicin-resistant cells derived from K562 and HL60 cells increased the sensitivity of both lines to doxorubicin. The authors hypothesize that correction of changes in the expression of these miRNAs could lead to overcoming resistance [164].

Downregulated miRNA-137 was found in adriamycin-resistant variants of MCF7 cells (MCF7/ADM), and its expression was inversely correlated with the expression of YB-1 (Y-box-1 binding protein) and P-gp [61]. MiRNA-137 appears to target YB-1, and its overexpression reduces the levels of both YB-1 and P-gp, thereby contributing to the MDR development of these cells. The authors also hypothesize that assisted elevation of these miRNA levels could contribute to the reversal of MDR [61].

P-gp is also an inversely regulated miRNA-491-3p that directly affects the transcription factor Sp3 (active in regulating P-gp transcription [165]) in doxorubicin- and vinblastine-resistant hepatocellular carcinoma cells [166]. This regulatory axis of miRNA-491-3p/Sp3/P-gp has also been confirmed in tissues from cancer patients.

Transfection of vincristine-resistant variant HL60 cells (HL60/VCR) with miRNA-138 resulted in decreased expression of P-gp and the antiapoptotic BCL-2 protein accompanied by increased expression of the proapoptotic BAX protein [167]. Cotransfection of the luciferase gene-conjugated P-gp promoter vector construct together with antagomirs of miRNA-138 (miRNA-138 antagonist) at various amounts indicated a concentration-dependent decrease in luciferase reporter activity, suggesting the effect of miRNA-138 on P-gp expression [167].

Additionally, miRNA-298 reduced P-gp expression probably by binding to the 3′-UTR of P-gp and suppressed doxorubicin resistance in breast cancer cells [168].

MiRNA-381 and miRNA-495 were downregulated in adriamycin-resistant cell variants of K562 cells [169]. Both miRNAs were targeted by the 3′-UTR of the ABCB1 gene, and the restoration of their expression in cells led to a decrease in P-gp expression at both the mRNA and protein levels and an increase in drug retention in the cell [169].

In 2014, a functional miRNA-508-5p was revealed by functional screening, the overexpression of which was able to reverse the resistance of gastric cancer cells to various chemotherapeutics in vitro and to increase the sensitivity of tumors to chemotherapy in vivo [170]. Further studies have shown that miRNA-508-5p could directly target the 3′-UTR of P-gp and domain containing zinc ribbon 1 (ZNRD1) and suppress their expression at both the mRNA and protein levels. The miRNA-508-5p/ZNRD1/P-gp regulatory loop plays a key role in MDR in gastric cancer. This miRNA could be used as a prognostic factor in gastric cancer [170].

#### 4.3.2. Breast Cancer Resistance Protein BCRP/ABCG2

BCRP is a half-transporter that contains only one ATP-binding domain with ABC consensus and one transmembrane domain that consists of six α-helical spans [171]. The fact that the ATP-binding domain is located closer to the N-terminus and the transmembrane domain to the C-terminus is a typical feature of the G subfamily of ABC transporters, making them different from members of other subfamilies; they are also referred to as transporters with reversed structure [172]. The significance of this reverse orientation for transporter function is not yet known [173]. Homodimerization of two BCRP molecules in the membrane allows the formation of a functional transporter. BCRP overexpression in breast cancer is associated with resistance to mitoxantrone (which is considered to be its prototypical substrate) and to topotecan, 7-ethyl-10-hydroxycamptothecin, anthracycline, and tamoxifen [174]. BCRP was the first transporter to confirm the regulation of its expression at the post-transcriptional level of miRNA [175].

Overexpression of miRNA-328 downregulates BCRP in breast cancer cells, which is associated with an increase in their sensitivity to mitoxantrone [176,177]. To et al. identified a binding site for miRNA-519c in the 3′-UTR of the ABCG2 gene [175]. Interestingly, compared to parental colon carcinoma S1 cells, their mitoxantrone-resistant variant S1MI80 cells lost miRNA-519c-mediated post-transcriptional control. This became a specific truncation of the A’GG 3′-UTR of the ABCG2 gene [175]. However, the miRNA-520h binding site, located 5’ upstream of the miRNA-519c binding site, was not affected by this truncation of the ABCG2 3′-UTR. Moreover, miRNA-520 h expression was lower in resistant cells than in parental cells, revealing another mechanism that increases BCRP expression and promotes the development of chemoresistance [175,178]. MiRNA-520h targets BCRP mRNA by RNA interference and is involved in hematopoietic stem cell differentiation [179] and increased BCRP expression, migration and invasion of pancreatic cancer cells [180].

Other miRNAs (miRNA-212, miRNA-181, and miRNA-487a) induced similar effects on BCRP expression [181,182,183,184].

In contrast, miRNA-132 is an indirect regulator of BCRP, which modulates cisplatin resistance by targeting the SIRT1 gene in gastric cancer stem cells [185]. SIRT1 is an upstream regulator of BCRP, and the authors have shown an inverse correlation between miRNA-132 and SIRT1.

Overexpression of miRNA-3163 significantly reduced BCRP expression in retinoblastoma stem cells and elevated cisplatin sensitivity [186].

#### 4.3.3. Multidrug Resistance-Associated Protein MRP1/ABCC1

MRP1 contains two ATP-binding sites and two standard transmembrane domains consisting of six α-helical spans. A special feature of this protein is the presence of another transmembrane domain located at the N-terminus, which consists of 5 α-helical spans [187]. The function of this additional transmembrane domain is unknown.

MRP1 is overexpressed in etoposide-resistant (VP16) cell variants of MCF-7/VP-16 compared to the parental MCF7 line [188]. Downregulation of miRNA-326 was observed in MCF7/VP-16 cells compared to MCF7 cells. Transfection of MCF7/VP-16 cells with miRNA-326 mimicking downregulated MRP1 increased the sensitivity of these cells to VP-16 and doxorubicin but not to mitoxantrone [188]. Another miRNA capable of directly downregulating MRP1 is miRNA-1291, which reduced the sensitivity of cancer cells to doxorubicin in pancreatic, lung, and kidney cancer [189].

Zhan et al. [190] showed that miRNA-145 regulated the resistance of gallbladder cancer cells to cisplatin by targeting MRP1. Low levels of miRNA-145 and overexpression of MRP1 in gallbladder tissue predicted a poor prognosis for gallbladder cancer patients receiving chemotherapy.

### 4.4. Effects of miRNAs on Cell Cycle Progression and Apoptosis Induction

Regulation of cell cycle progression plays an important role in the resistance of cancer cells to chemotherapeutics. The central tumor suppressor p53 is a multilevel regulator of cellular processes that also plays a key role in cell cycling regulation. As a transcription factor, p53 induces the expression of the p21 inhibitors CDK1 and CDK2 and the subsequent cell cycle arrest [191]. In addition, p53 also induces the expression of the proapoptotic protein BAX and is therefore co-responsible for whether cell cycle arrest goes into apoptosis. The transcription factor p53, which also controls the expression of genes associated with the onset and progression of cell death, is a key player in controlling the cell survival/cell death balance [192,193,194]. As a transcription factor, it also plays an important role in regulating miRNA biogenesis. When DNA damage occurs in cells due to tumor transformation, p53 acts as a tumor suppressor, and its expression/stabilization in cells is increased. An additional increase in p53 expression could be induced by members of the miRNA-34 family (miRNA-34a, miRNA-34b, and miRNA-34c) [195]. In particular, miRNA-34a, as well as other members of the miRNA-34 family, are considered to be tumor suppressor miRNAs and can improve the sensitivity of tumor cells to chemotherapeutic drugs [196]. Conversely, p53 directly recognizes miRNA-34 family promoters and activates their transcription [197], inducing the expression of other miRNAs, such as miRNA-145, miRNA-107, miRNA-192, and miRNA-215 [198].

Several lines of evidence show that there are two other transcription factors, p63 and p73, which are similar to p53, and all three are members of the p53 family [199]. These three transcription factors may play different roles in cells, but p63 and p73 appear to coordinate p53 in balancing cell survival, cell death, and cell senescence. Ory et al. [200] found that p63 and p73 can regulate miRNA-193a-5p expression. While p63 is a transcriptional repressor of miRNA-193a, p73 functions as a direct transcriptional activator of this miRNA. Excess miRNA-193a suppresses p73 function via feedback inhibition [42]. Cisplatin treatment leads to the degradation of p63 and the activation of p73, which together induce miRNA-193a transcription. However, as the cellular content of miRNA-193a increases, p73 cellular function is suppressed, and the therapeutic response to cisplatin is reduced. Importantly, elimination of miRNA-193a function disrupted this feedback, thereby suppressing tumor cell viability and inducing significant cell sensitivity to cisplatin in vitro and in vivo [200].

The expression of p53 is controlled at the post-transcriptional level by multiple miRNAs, some of which are exemplified. Upregulation of miRNA-125b suppresses the expression of p53 and the proapoptotic protein BAK, thereby preventing the induction of p53-dependent apoptosis and causing chemoresistance to doxorubicin, vincristine, and etoposide in EWS (Ewing sarcoma/primitive neuroectodermal tumor) cells [201].

The interaction of miRNA-122 and cyclin G1 positively regulates the stability and transcriptional activity of p53 and affects the sensitivity of human hepatocarcinoma cells to doxorubicin [202]. MiRNA-34a is one of the effector genes of p53, and its expression linearly correlates with the expression of p53 [203]. Overexpression of miRNA-34a seems to inhibit cell growth and induce apoptosis by targeting CDK6 (cyclin independent kinase 6) [204].

MiRNA-140 regulates chemoresistance by inducing G1/G2 phase cell arrest mediated in part by the inhibition of HDAC4 expression in colon cancer cells. Downregulation of HDAC4 may contribute to the induction of p21 in the presence of unmutated p53 [205]. Zhang et al. [206] identified a novel p53/miRNA-520g/p21 signaling pathway that affects the response of colon cancer cells to fluorouracil and oxaliplatin. MiR-520g mediated this drug resistance via direct targeting of p21 expression. Moreover, p53 suppressed miRNA-520g expression antagonizes its effects.

MiRNA-302b reduces the resistance of breast cancer cells to cisplatin by targeting E2F1, which is the major regulator of the G1 to S phase cell transition [207]. E2F1 transcriptionally activates/induces the expression of ATM (the major sensor of DNA damage in the cell). Through the downregulation of E2F1, miRNA-302b indirectly affects ATM expression and prevents cell cycle progression after cisplatin treatment. It also impairs the ability of cells to repair damaged DNA and increases the activation of apoptosis [207]. In tamoxifen-resistant breast cancer cells, miRNA-221/222 has been shown to target another cell cycle inhibitor, p27, which contributes to antagonizing cell death and promotes hormone-independent cell growth [208].

Restoration of miRNA-320a expression increased the susceptibility of tamoxifen-resistant BCa cells by reducing the regulation of c-Myc and cyclin D1 [209].

MiRNA-25 appears to be overexpressed in most cases of lung cancer [210]. Downregulation of miRNA-25 in the H510A cell line reduced its proliferation, invasiveness, and resistance to cisplatin. It also induced G0/G1 cell arrest by downregulating cyclin E2 and CDK2. Cyclin E2 overexpression in H510A cells reversed cell cycle arrest and restored invasiveness, which was disrupted by the downregulation of miRNA-25 [210]. Inhibition of cell proliferation has also been described for miRNA-31, and its expression in the cell adversely affects the expression of many factors involved in maintaining DNA replication as well as the prosurvival phosphatase PPP6C, which is associated with resistance to chemotherapy and radiation therapy [211].

Apoptosis is a tightly regulated process that removes damaged cells that are unable to maintain coordinated homeostasis with neighboring cells in individual tissues. When the apoptosis process fails, damaged cells can begin to divide uncontrollably and form tumors. Due to the rapid proliferation of such damaged cells, they have accelerated metabolisms and are more easily affected by cytotoxic substances. To eliminate such cells, effective chemotherapy is used, which forces them to enter the processes of cell death. However, during treatment, cells may become resistant to cytostatics by altering the cellular contents of proapoptotic/antiapoptotic proteins of the BCL-2 [212] family, tumor suppressor genes [213], oncogenes (Ras, myc [214]), and endoplasmic reticulum stress-related genes (*GRP78*, *XBP1*, and *GRP94* [154]), which can regulate miRNAs.

BCL-2 is a key inhibitor of outer mitochondrial membrane permeabilization by proapoptotic proteins and inhibits the release of cytochrome c from mitochondria [215]. Cytochrome c in the cytosol induces the activation of initiating caspases and apoptosis.

Calin et al. [216] demonstrated decreased expression of miRNA-15a and miRNA-16-1 in patients with chronic lymphocytic leukemia. Both miRNA-15a and miRNA-16-1 are involved in the downregulation of the expression of the antiapoptotic gene BCL-2 [217,218], which is overexpressed in tumors, and also due to the reduced function of both miRNAs. BCL-2 overexpression has been observed in some types of cancer, including leukemia, lymphoma, carcinomas, and breast cancer. Downregulated miRNA-15b and miRNA-16 and upregulated BCL-2 protein were found by Xia et al. [219] in resistant gastric cancer cells, which was accompanied by increased sensitivity of the cells to chemotherapeutics. Gemcitabine resistance in pancreatic cancer cells was mediated by miRNA-21 by direct upregulation of BCL-2 and downregulation of BAX. MiRNA21 promotes BCL-2 expression by directly binding to the 3’UTR of its mRNA [220]. Singh et al. [221] showed that overexpression of miRNA-195, miRNA-24-2, and miRNA-365-2 impaired the antiapoptotic activity of BCL-2 observed in HEK-293T and MCF7 cells, leading to increased induction of etoposide-induced apoptosis. In vitro and in vivo gain- or loss-of-function experiments in drug-resistant gastric cancer suggest that miRNA-495-3p directly affects the molecular chaperone GRP78, which alters the balance between cellular autophagy and apoptosis in MDR cells. [222]. The authors suggest that GRP78, as a regulator of autophagy, could prevent MDR cells from entering the process of apoptosis. At the same time, this miRNA reduces the cellular content of BCL-2 and, conversely, increases BAX, leading to the release of cytochrome c into the cytosol, activation of initiating caspase, and apoptosis [222]. These miRNAs may be potential therapeutic targets due to their proapoptotic roles in cancer cells. In gastric cancer cell lines, artificial expression of miRNA-204 correlates with decreased BCL-2 regulation and inhibits colony formation and tumor cell migration [223]. MiRNA-204 increases the sensitivity of gastric cancer cells to 5-fluorouracil and oxaliplatin.

MiRNAs can also regulate apoptosis by targeting other members of the BCL-2 family of proteins. For example, the antiapoptotic protein BCL-XL may be downregulated by miRNA-574-3p [224]. MiRNA-101 targets MCL-1, reduces its expression and increases the sensitivity of hepatocellular carcinoma cells to doxorubicin-induced apoptosis [225].

Pro-apoptotic proteins of the BCL-2 family may also be targeted by miRNAs. For example, miRNA-494 can induce TRAIL (TNF-related apoptosis-inducing ligand) resistance in non-small cell lung cancer through the downregulation of BIM [226]. Upregulation of miRNA-365 can induce gemcitabine resistance by downregulating the expression of the proapoptotic protein BAX [227].

## 5. Conclusions

Although significant progress has been made in recent decades in understanding the role of miRNAs in the development of multidrug resistance in neoplastic diseases, it is still very difficult to compile a complete summary of the regulatory pathways involved in these events. We have tried to provide a clear summary of knowledge about the possible roles of miRNAs in the development of the most common mechanisms of MDR. Based on these findings, we note that miRNAs may play a key and possibly predominant role in the development of the MDR phenotype. This suggests that understanding the effect of miRNAs in the development of MDR in specific cases will enable their use as important prognostic markers in the diagnosis and/or promising therapeutic targets for effective treatment.

It is thought that miRNAs could be a suitable therapeutic tool in the treatment of cancer. Their use is hindered by problems in the delivery of miRNAs to the site of action, their limited stability, and the possibility of off-target effects [228]. These limitations could be overcome by using appropriate carriers that would target miRNAs or their precursors directly to target cells. It appears that a possible vehicle for miRNAs could be exosomes formed and secreted by mesenchymal stem cells [229]. Further oriented research is needed to meet this goal.

The possibility of modulating miRNA expression and function as a therapeutic strategy is also interesting. Correction of miRNA expression in neoplastic cells to approximate the profile of normal cells appears to be desirable. However, the regulation of cellular miRNA content is a very complex mechanism, the individual parts of which have not yet been fully elucidated. Several lines of evidence suggest that exosomes and other extracellular vesicles secreted by cancer cells are involved in the development and metastasis of cancer (reviewed in [230]). Tumor cells that have undergone epithelial–mesenchymal transition play an important role here [231]. Influencing these processes could also bring significant progress in the targeted treatment of neoplastic diseases. Therefore, we emphasize the need for further research aimed at elucidating in detail the fine regulation of miRNA production and degradation.

## Supplementary Materials

The following supporting information can be downloaded at: https://www.mdpi.com/article/10.3390/cancers14041090/s1: Figure S1: The balance between cell death stimuli and cell survival stimuli is important in relation to tumor cell survival and their sensitivity to drug therapy; Table S1: Effects of miRNA on proteins responsible drug resistance.
