# Supplementary material for: The Roles of microRNAs in Cancer Multidrug Resistance
Source: Cancers (Basel). 2022 Feb 21;14(4):1090. doi: 10.3390/cancers14041090 (PMC8870231; doi:10.3390/cancers14041090)
Supplement: Supplementary file 1 [file cancers-14-01090-s001.zip › cancers-1598840-supplementary.pdf]

Supplementary Files

The roles of microRNAs in cancer multidrug resistance

Lucia Pavlicova et al.

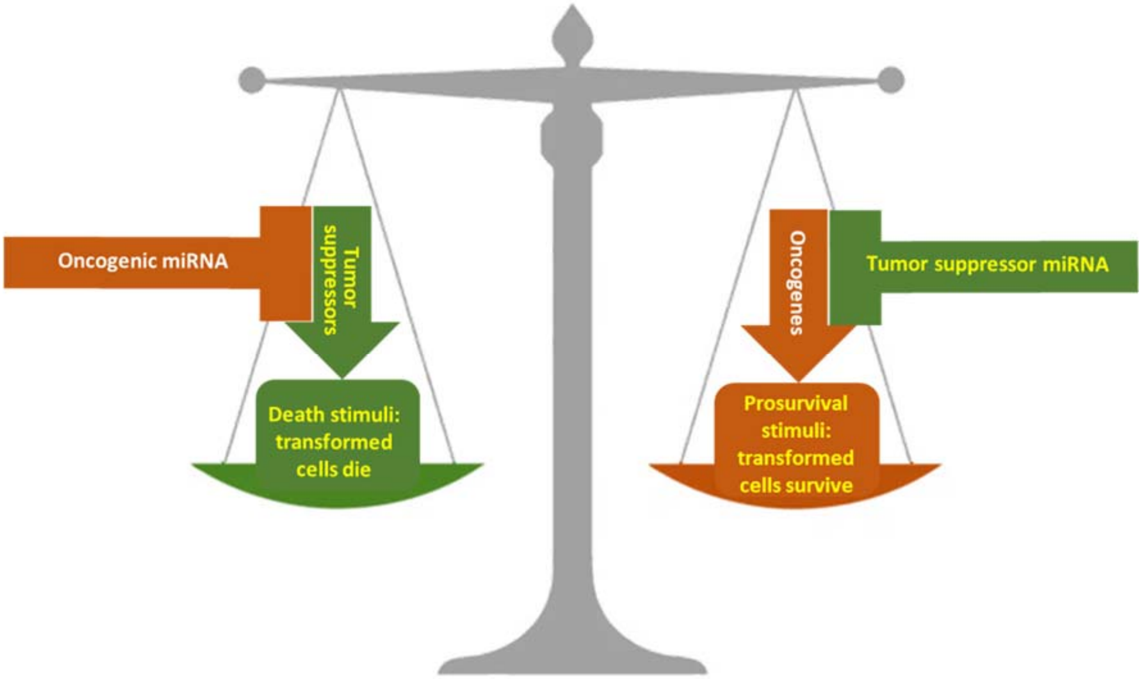

Figure S1. The balance between cell death stimuli and cell survival stimuli is important in relation to tumor cell survival and their sensitivity to drug therapy. After damage to a cell that leads to the initiation of its tumor transformation, cell death programs can be triggered through the expression of tumor suppressor genes (cell death stimuli) and the cell is eliminated. Oncogenic miRNAs, which suppress the expression of tumor suppressor genes, may counteract this [1,2]. Oncogenes (eg, antiapoptotic proteins), the expression of which is a stimulus for cell survival, prevent the initiation of cell death processes. Oncogene expression is attenuated by tumor suppressor miRNAs [1,2]. Thus, the fate of cells that have entered the process of carcinogenesis also depends on the balance of oncogenic and tumor suppressor miRNAs.

Table S1: Effects of miRNA on expression of proteins responsible drug resistance.
Section A – Effect of miRNA on the expression of proteins providing 1st, 2nd and 3rd phase of cell detoxification

1. Phase	Oxidizing cytochrom P450 enzymes		
miRNA	Target Gene	↑ upregulation<br>↓ downregulation	Citation
miRNA-132-5p	CYP1A2	↓	[3]
miRNA-200c<br>miRNA-27b	CYP1B1	↓<br>↓	[4]<br>[5,6]
miRNA-206<br>miRNA-148a<br>miRNA-27b	CYP3A4	↓<br>↓<br>↓	[7,8]<br>[7,8]<br>[5,6]
let-7b	CYP2J2	↓	[9]
2. Phase	Conjugating enzymes		
miRNA-631	SULT1A1	↓	[10]
miRNA-133a<br>miRNA-513a-3p	GSTP1	↓<br>↓	[11,12]<br>[11,12]
miRNA-133b	GSTπ	↓	[13]
3. Phase	Membráne efflux transporters		
miRNA-451<br>miRNA-27a<br>miRNA-27a<br>miRNA-331-5p<br>miRNA-137<br>miRNA-491-3p<br>miRNA-138<br>miRNA-298<br>miRNA-508-5p<br>miRNA-19a<br>miRNA-19b<br>miRNA-130a<br>miRNA-205<br>miRNA-214<br>miRNA-495<br>miRNA-381	P-gp	↑↓<br>↑<br>↓<br>↓<br>↓<br>↓<br>↓<br>↓<br>↓<br>↑<br>↑<br>↑<br>↓<br>↓<br>↓<br>↓	[14,15]<br>[15]<br>[16-18]<br>[17]<br>[19]<br>[20]<br>[21]<br>[22]<br>[23]<br>[24]<br>[24]<br>[25]<br>[26]<br>[27]<br>[14]<br>[14]
miRNA-519c<br>miRNA-520a<br>miRNA-212<br>miRNA-3136<br>miRNA-181a<br>miRNA-487a<br>miRNA-132	BCRP	↓<br>↓<br>↓<br>↓<br>↓<br>↓<br>↓	[28-30]<br>[28-30]<br>[31]<br>[32]<br>[33]<br>[34]<br>[35]
miRNA-326<br>miRNA-1291<br>miRNA-145	MRP1	↓<br>↓<br>↓	[36]<br>[37]<br>[38]

Section B – Effect of miRNA on the expression of proteins active in cell cycling progression.

miRNA	Target Gene	↑ upregulation<br>↓ downregulation	Citation
miRNA-125b<br>miRNA-34a	p53	↓<br>↓	[38-40]<br>[38-40]
miRNA-140	HDAC4	↓	[41]
miRNA-302b	E2F1	↓	[42]
miRNA-25	cyclin E2<br>CDK2	↑<br>↑	[43]
miRNA-122	cyclin G1	↓	[44]
miRNA-320a	cMyc<br>cyclin D1	↓<br>↓	[45]
miRNA-520g<br>miRNA-140	p21	↓<br>↑	[46,47]<br>[41]
miRNA-221/222	p27	↓	[46,47]
miRNA-31	PPP6C	↓	[48]

Section C – Effect of miRNA on the expression of proteins regulating apoptosis

miRNA	Target Gene	↑ upregulation<br>↓ downregulation	Citation
miRNA-15b<br>miRNA-16<br>miRNA-21<br>miRNA-195<br>miRNA-24-2<br>miRNA-365-2<br>miRNA-204<br>miRNA-138<br>miRNA-495-3p	BCL2	↑<br>↑<br>↑<br>↓<br>↓<br>↓<br>↓<br>↓<br>↓	[49]<br>[49]<br>[50]<br>[51,52]<br>[51,52]<br>[51,52]<br>[51,52]<br>[21]<br>[53]
miRNA-21	BAX	↓	[50]
miRNA-574-3p	BCL-XL	↓	[54]
miRNA-101	MCL-1	↓	[53]
miRNA-494	BIM	↓	[55]
miRNA-365<br>miRNA-138	BAX	↓<br>↑	[56]<br>[21]
miRNA-125b	BAK	↓	[40]
miRNA-495-3p	GRP78	↓	[57]

References

1. Gambari, R.; Brognara, E.; Spandidos, D.A.; Fabbri, E. Targeting oncomirnas and mimicking tumor suppressor mirnas: Nuew trends in the development of mirna therapeutic strategies in oncology (review). Int J Oncol 2016, 49, 5-32.
2. Lotterman, C.D.; Kent, O.A.; Mendell, J.T. Functional integration of micrornas into oncogenic and tumor suppressor pathways. Cell Cycle 2008, 7, 2493-2499.
3. Chen, Y.; Zeng, L.; Wang, Y.; Tolleson, W.H.; Knox, B.; Chen, S.; Ren, Z.; Guo, L.; Mei, N.; Qian, F., et al. The expression, induction and pharmacological activity of cyp1a2 are post-transcriptionally regulated by microrna hsa-mir-132-5p. Biochem Pharmacol 2017, 145, 178-191.
4. Chang, I.; Mitsui, Y.; Fukuhara, S.; Gill, A.; Wong, D.K.; Yamamura, S.; Shahryari, V.; Tabatabai, Z.L.; Dahiya, R.; Shin, D.M., et al. Loss of mir-200c up-regulates cyp1b1 and confers docetaxel resistance in renal cell carcinoma. Oncotarget 2015, 6, 7774-7787.
5. Martinez, V.G.; O'Connor, R.; Liang, Y.; Clynes, M. Cyp1b1 expression is induced by docetaxel: Effect on cell viability and drug resistance. Br J Cancer 2008, 98, 564-570.
6. Tsuchiya, Y.; Nakajima, M.; Takagi, S.; Taniya, T.; Yokoi, T. Microrna regulates the expression of human cytochrome p450 1b1. Cancer Res 2006, 66, 9090-9098.
7. Liu, J.E.; Ren, B.; Tang, L.; Tang, Q.J.; Liu, X.Y.; Li, X.; Bai, X.; Zhong, W.P.; Meng, J.X.; Lin, H.M., et al. The independent contribution of mirnas to the missing heritability in cyp3a4/5 functionality and the metabolism of atorvastatin. Sci Rep 2016, 6, 26544.
8. Takagi, S.; Nakajima, M.; Mohri, T.; Yokoi, T. Post-transcriptional regulation of human pregnane x receptor by micro-rna affects the expression of cytochrome p450 3a4. J Biol Chem 2008, 283, 9674-9680.
9. Chen, F.; Chen, C.; Yang, S.; Gong, W.; Wang, Y.; Cianflone, K.; Tang, J.; Wang, D.W. Let-7b inhibits human cancer phenotype by targeting cytochrome p450 epoxygenase 2j2. PLoS One 2012, 7, e39197.
10. Yu, X.; Dhakal, I.B.; Beggs, M.; Edavana, V.K.; Williams, S.; Zhang, X.; Mercer, K.; Ning, B.; Lang, N.P.; Kadlubar, F.F., et al. Functional genetic variants in the 3'-untranslated region of sulfotransferase isoform 1a1 (sult1a1) and their effect on enzymatic activity. Toxicol Sci 2010, 118, 391-403.
11. Moriya, Y.; Nohata, N.; Kinoshita, T.; Mutallip, M.; Okamoto, T.; Yoshida, S.; Suzuki, M.; Yoshino, I.; Seki, N. Tumor suppressive microrna-133a regulates novel molecular networks in lung squamous cell carcinoma. J Hum Genet 2012, 57, 38-45.
12. Zhang, X.; Zhu, J.; Xing, R.; Tie, Y.; Fu, H.; Zheng, X.; Yu, B. Mir-513a-3p sensitizes human lung adenocarcinoma cells to chemotherapy by targeting gstp1. Lung Cancer 2012, 77, 488-494.
13. Chen, S.; Jiao, J.W.; Sun, K.X.; Zong, Z.H.; Zhao, Y. Microrna-133b targets glutathione s-transferase pi expression to increase ovarian cancer cell sensitivity to chemotherapy drugs. Drug Des Devel Ther 2015, 9, 5225-5235.
14. Xu, Y.; Ohms, S.J.; Li, Z.; Wang, Q.; Gong, G.; Hu, Y.; Mao, Z.; Shannon, M.F.; Fan, J.Y. Changes in the expression of mir-381 and mir-495 are inversely associated with the expression of the mdr1 gene and development of multi-drug resistance. PLoS One 2013, 8, e82062.
15. Zhu, H.; Wu, H.; Liu, X.; Evans, B.R.; Medina, D.J.; Liu, C.G.; Yang, J.M. Role of microrna mir-27a and mir-451 in the regulation of mdr1/p-glycoprotein expression in human cancer cells. Biochem Pharmacol 2008, 76, 582-588.
16. Chen, Z.; Ma, T.; Huang, C.; Zhang, L.; Lv, X.; Xu, T.; Hu, T.; Li, J. Mir-27a modulates the mdr1/p-glycoprotein expression by inhibiting fzd7/beta-catenin pathway in hepatocellular carcinoma cells. Cell Signal 2013, 25, 2693-2701.
17. Feng, D.D.; Zhang, H.; Zhang, P.; Zheng, Y.S.; Zhang, X.J.; Han, B.W.; Luo, X.Q.; Xu, L.; Zhou, H.; Qu, L.H., et al. Down-regulated mir-331-5p and mir-27a are associated with chemotherapy resistance and relapse in leukaemia. J Cell Mol Med 2011, 15, 2164-2175.
18. Messingerova, L.; Imrichova, D.; Kavcova, H.; Seres, M.; Sulova, Z.; Breier, A. A decrease in cellular microrna-27a content is involved in azacytidine-induced p-glycoprotein expression in skm-1 cells. Toxicol In Vitro 2016, 36, 81-88.
19. Zhu, X.; Li, Y.; Shen, H.; Li, H.; Long, L.; Hui, L.; Xu, W. Mir-137 restoration sensitizes multidrug-resistant mcf-7/adm cells to anticancer agents by targeting yb-1. Acta Biochim Biophys Sin (Shanghai) 2013, 45, 80-86.
20. Zhao, Y.; Qi, X.; Chen, J.; Wei, W.; Yu, C.; Yan, H.; Pu, M.; Li, Y.; Miao, L.; Li, C., et al. The mir-491-3p/sp3/abcb1 axis attenuates multidrug resistance of hepatocellular carcinoma. Cancer Lett 2017, 408, 102-111.
21. Zhao, X.; Yang, L.; Hu, J.; Ruan, J. Mir-138 might reverse multidrug resistance of leukemia cells. Leuk Res 2010, 34, 1078-1082.
22. Bao, L.; Hazari, S.; Mehra, S.; Kaushal, D.; Moroz, K.; Dash, S. Increased expression of p-glycoprotein and doxorubicin chemoresistance of metastatic breast cancer is regulated by mir-298. Am J Pathol 2012, 180, 2490-2503.

23. Shang, Y.; Zhang, Z.; Liu, Z.; Feng, B.; Ren, G.; Li, K.; Zhou, L.; Sun, Y.; Li, M.; Zhou, J., et al. Mir-508-5p regulates multidrug resistance of gastric cancer by targeting abcb1 and znrd1. Oncogene 2014, 33, 3267-3276.
24. Wang, F.; Li, T.; Zhang, B.; Li, H.; Wu, Q.; Yang, L.; Nie, Y.; Wu, K.; Shi, Y.; Fan, D. Microrna-19a/b regulates multidrug resistance in human gastric cancer cells by targeting pten. Biochem Biophys Res Commun 2013, 434, 688-694.
25. Li, N.; Yang, L.; Wang, H.; Yi, T.; Jia, X.; Chen, C.; Xu, P. Mir-130a and mir-374a function as novel regulators of cisplatin resistance in human ovarian cancer a2780 cells. PLoS One 2015, 10, e0128886.
26. Nagesh, P.K.B.; Chowdhury, P.; Hatami, E.; Boya, V.K.N.; Kashyap, V.K.; Khan, S.; Hafeez, B.B.; Chauhan, S.C.; Jaggi, M.; Yallapu, M.M. Mirna-205 nanoformulation sensitizes prostate cancer cells to chemotherapy. Cancers (Basel) 2018, 10.
27. Jin, J.; Yao, J.; Yue, F.; Jin, Z.; Li, D.; Wang, S. Decreased expression of microrna-214 contributes to imatinib mesylate resistance of chronic myeloid leukemia patients by upregulating abcb1 gene expression. Exp Ther Med 2018, 16, 1693-1700.
28. Li, X.; Pan, Y.Z.; Seigel, G.M.; Hu, Z.H.; Huang, M.; Yu, A.M. Breast cancer resistance protein bcrp/abcg2 regulatory micrornas (hsa-mir-328, -519c and -520h) and their differential expression in stem-like abcg2+ cancer cells. Biochem Pharmacol 2011, 81, 783-792.
29. To, K.K.; Robey, R.W.; Knutsen, T.; Zhan, Z.; Ried, T.; Bates, S.E. Escape from hsa-mir-519c enables drug-resistant cells to maintain high expression of abcg2. Mol Cancer Ther 2009, 8, 2959-2968.
30. To, K.K.; Zhan, Z.; Litman, T.; Bates, S.E. Regulation of abcg2 expression at the 3' untranslated region of its mrna through modulation of transcript stability and protein translation by a putative microrna in the s1 colon cancer cell line. Mol Cell Biol 2008, 28, 5147-5161.
31. Turrini, E.; Haenisch, S.; Laechelt, S.; Diewock, T.; Bruhn, O.; Cascorbi, I. Microrna profiling in k-562 cells under imatinib treatment: Influence of mir-212 and mir-328 on abcg2 expression. Pharmacogenet Genomics 2012, 22, 198-205.
32. Jia, M.; Wei, Z.; Liu, P.; Zhao, X. Silencing of abcg2 by microrna-3163 inhibits multidrug resistance in retinoblastoma cancer stem cells. J Korean Med Sci 2016, 31, 836-842.
33. Jiao, X.; Zhao, L.; Ma, M.; Bai, X.; He, M.; Yan, Y.; Wang, Y.; Chen, Q.; Zhao, X.; Zhou, M., et al. Mir-181a enhances drug sensitivity in mitoxantone-resistant breast cancer cells by targeting breast cancer resistance protein (bcrp/abcg2). Breast Cancer Res Treat 2013, 139, 717-730.
34. Ma, M.T.; He, M.; Wang, Y.; Jiao, X.Y.; Zhao, L.; Bai, X.F.; Yu, Z.J.; Wu, H.Z.; Sun, M.L.; Song, Z.G., et al. Mir-487a resensitizes mitoxantrone (mx)-resistant breast cancer cells (mcf-7/mx) to mx by targeting breast cancer resistance protein (bcrp/abcg2). Cancer Lett 2013, 339, 107-115.
35. Zhang, L.; Guo, X.; Zhang, D.; Fan, Y.; Qin, L.; Dong, S.; Zhang, L. Upregulated mir-132 in lgr5(+) gastric cancer stem cell-like cells contributes to cisplatin-resistance via sirt1/creb/abcg2 signaling pathway. Mol Carcinog 2017, 56, 2022-2034.
36. Liang, Z.; Wu, H.; Xia, J.; Li, Y.; Zhang, Y.; Huang, K.; Wagar, N.; Yoon, Y.; Cho, H.T.; Scala, S., et al. Involvement of mir-326 in chemotherapy resistance of breast cancer through modulating expression of multidrug resistance-associated protein 1. Biochem Pharmacol 2010, 79, 817-824.
37. Pan, Y.Z.; Zhou, A.; Hu, Z.; Yu, A.M. Small nucleolar rna-derived microrna hsa-mir-1291 modulates cellular drug disposition through direct targeting of abc transporter abcc1. Drug Metab Dispos 2013, 41, 1744-1751.
38. Zhan, M.; Zhao, X.; Wang, H.; Chen, W.; Xu, S.; Wang, W.; Shen, H.; Huang, S.; Wang, J. Mir-145 sensitizes gallbladder cancer to cisplatin by regulating multidrug resistance associated protein 1. Tumour Biol 2016, 37, 10553-10562.
39. Fujita, Y.; Kojima, K.; Hamada, N.; Ohhashi, R.; Akao, Y.; Nozawa, Y.; Deguchi, T.; Ito, M. Effects of mir-34a on cell growth and chemoresistance in prostate cancer pc3 cells. Biochem Biophys Res Commun 2008, 377, 114-119.
40. Iida, K.; Fukushi, J.; Matsumoto, Y.; Oda, Y.; Takahashi, Y.; Fujiwara, T.; Fujiwara-Okada, Y.; Hatano, M.; Nabashima, A.; Kamura, S., et al. Mir-125b develops chemoresistance in ewing sarcoma/primitive neuroectodermal tumor. Cancer Cell Int 2013, 13, 21.
41. Song, B.; Wang, Y.; Xi, Y.; Kudo, K.; Bruheim, S.; Botchkina, G.I.; Gavin, E.; Wan, Y.; Formentini, A.; Kornmann, M., et al. Mechanism of chemoresistance mediated by mir-140 in human osteosarcoma and colon cancer cells. Oncogene 2009, 28, 4065-4074.
42. Cataldo, A.; Cheung, D.G.; Balsari, A.; Tagliabue, E.; Coppola, V.; Iorio, M.V.; Palmieri, D.; Croce, C.M. Mir-302b enhances breast cancer cell sensitivity to cisplatin by regulating e2f1 and the cellular DNA damage response. Oncotarget 2016, 7, 786-797.
43. Zhao, Z.; Liu, J.; Wang, C.; Wang, Y.; Jiang, Y.; Guo, M. Microrna-25 regulates small cell lung cancer cell development and cell cycle through cyclin e2. Int J Clin Exp Pathol 2014, 7, 7726-7734.
44. Fornari, F.; Gramantieri, L.; Giovannini, C.; Veronese, A.; Ferracin, M.; Sabbioni, S.; Calin, G.A.; Grazi, G.L.; Croce, C.M.; Tavolari, S., et al. Mir-122/cyclin g1 interaction modulates p53 activity and affects doxorubicin sensitivity of human hepatocarcinoma cells. Cancer Res 2009, 69, 5761-5767.

45. Lu, M.; Ding, K.; Zhang, G.; Yin, M.; Yao, G.; Tian, H.; Lian, J.; Liu, L.; Liang, M.; Zhu, T., et al. Microrna-320a sensitizes tamoxifen-resistant breast cancer cells to tamoxifen by targeting arpp-19 and errgamma. Sci Rep 2015, 5, 8735.
46. Miller, T.E.; Ghoshal, K.; Ramaswamy, B.; Roy, S.; Datta, J.; Shapiro, C.L.; Jacob, S.; Majumder, S. Microrna-221/222 confers tamoxifen resistance in breast cancer by targeting p27kip1. J Biol Chem 2008, 283, 29897-29903.
47. Zhang, Y.; Geng, L.; Talmon, G.; Wang, J. Microrna-520g confers drug resistance by regulating p21 expression in colorectal cancer. J Biol Chem 2015, 290, 6215-6225.
48. Ivanov, S.V.; Goparaju, C.M.; Lopez, P.; Zavadil, J.; Toren-Haritan, G.; Rosenwald, S.; Hoshen, M.; Chajut, A.; Cohen, D.; Pass, H.I. Pro-tumorigenic effects of mir-31 loss in mesothelioma. J Biol Chem 2010, 285, 22809-22817.
49. Xia, L.; Zhang, D.; Du, R.; Pan, Y.; Zhao, L.; Sun, S.; Hong, L.; Liu, J.; Fan, D. Mir-15b and mir-16 modulate multidrug resistance by targeting bcl2 in human gastric cancer cells. Int J Cancer 2008, 123, 372-379.
50. Dong, J.; Zhao, Y.P.; Zhou, L.; Zhang, T.P.; Chen, G. Bcl-2 upregulation induced by mir-21 via a direct interaction is associated with apoptosis and chemoresistance in mia paca-2 pancreatic cancer cells. Arch Med Res 2011, 42, 8-14.
51. Sacconi, A.; Biagioni, F.; Canu, V.; Mori, F.; Di Benedetto, A.; Lorenzon, L.; Ercolani, C.; Di Agostino, S.; Cambria, A.M.; Germoni, S., et al. Mir-204 targets bcl-2 expression and enhances responsiveness of gastric cancer. Cell Death Dis 2012, 3, e423.
52. Singh, R.; Saini, N. Downregulation of bcl2 by mirnas augments drug-induced apoptosis--a combined computational and experimental approach. J Cell Sci 2012, 125, 1568-1578.
53. He, H.; Tian, W.; Chen, H.; Deng, Y. Microrna-101 sensitizes hepatocellular carcinoma cells to doxorubicin-induced apoptosis via targeting mcl-1. Mol Med Rep 2016, 13, 1923-1929.
54. Chiyomaru, T.; Yamamura, S.; Fukuhara, S.; Hidaka, H.; Majid, S.; Saini, S.; Arora, S.; Deng, G.; Shahryari, V.; Chang, I., et al. Genistein up-regulates tumor suppressor microrna-574-3p in prostate cancer. PLoS One 2013, 8, e58929.
55. Romano, G.; Acunzo, M.; Garofalo, M.; Di Leva, G.; Cascione, L.; Zanca, C.; Bolon, B.; Condorelli, G.; Croce, C.M. Mir-494 is regulated by erk1/2 and modulates trail-induced apoptosis in non-small-cell lung cancer through bim down-regulation. Proc Natl Acad Sci U S A 2012, 109, 16570-16575.
56. Hamada, S.; Masamune, A.; Miura, S.; Satoh, K.; Shimosegawa, T. Mir-365 induces gemcitabine resistance in pancreatic cancer cells by targeting the adaptor protein shc1 and pro-apoptotic regulator bax. Cell Signal 2014, 26, 179-185.
57. Chen, S.; Wu, J.; Jiao, K.; Wu, Q.; Ma, J.; Chen, D.; Kang, J.; Zhao, G.; Shi, Y.; Fan, D., et al. Microrna-495-3p inhibits multidrug resistance by modulating autophagy through grp78/mtor axis in gastric cancer. Cell Death Dis 2018, 9, 1070.
